# Impulsivity and Early Initiation of Sexual Behaviors in Adolescents with Externalizing Disorders

**DOI:** 10.1007/s10508-025-03305-0

**Published:** 2026-01-03

**Authors:** Paola P. Mattey-Mora, Trey V. Dellucci, Michael P. Smoker, Matthew C. Aalsma, Leslie A. Hulvershorn

**Affiliations:** 1 Department of Psychiatry, Indiana University School of Medicine, 410 W 10th Street, HS 2000, Indianapolis, IN 46202 USA; 2 Department of Pediatrics, Indiana University School of Medicine, Indianapolis, IN USA

**Keywords:** DSM-5, Externalizing disorders, Sexual initiation, Sexting, Sexual behaviors, Adolescents

## Abstract

Impulsivity, a multidimensional construct characterized by rash decision-making and difficulty delaying gratification, is a trait of externalizing disorders (e.g., attention-deficit/hyperactivity, disruptive, oppositional defiant, and conduct disorders) and has been associated with risk-taking behaviors. However, its association with risky sexual behaviors in this population remains limited. This observational study examined the association between impulsivity and early sexual behaviors in 96 adolescents (male *n* = 63) with externalizing disorders. Impulsivity at baseline was assessed using the Urgency-Premeditation-Perseverance-Sensation Seeking-Positive Urgency (UPPS-P) scale. Engagement in early sexual behaviors was categorized into three groups: sexting; other sexual behaviors (e.g., genital touch, oral sex); and sexual intercourse. Relative risk (RR) was estimated using unadjusted and adjusted (A) modified Poisson regressions with robust variance. Adjusted models included biological sex, family history of substance use, and parental education as potential confounders. Significant associations were found between sexting and both overall impulsivity (ARR = 1.11, 95% CI = 1.02–1.21) and sensation seeking (ARR = 1.45, 95% CI = 1.18–1.79). Sensation seeking was also significantly associated with other sexual behaviors (ARR = 1.44, 95% CI = 1.01–2.08). No significant associations were found between impulsivity subscales and sexual intercourse. These findings highlight the role of sensation seeking in early sexting and other sexual behaviors among adolescents with externalizing disorders. Interventions targeting sensation seeking may be beneficial for reducing sexual risk-taking in high-risk youth. Future studies are needed to understand the effects of impulsivity in risky sexual behaviors over time, particularly across late adolescence and young adulthood in this population.

## Introduction

Early sexual initiation, defined as first sexual intercourse before age 14 (Minnis et al., 2022), has been identified as a predictor of adverse outcomes, including unintended pregnancy (Prendergast et al., 2019), sexually transmitted infections (Epstein et al., 2014), increased number of sexual partners (Kugler et al., 2017), and psychosocial challenges such as depression and relationship power imbalances (Wesche et al., 2017). Over the past two decades, adolescent sexual behaviors have undergone significant shifts. The prevalence of adolescents engaging in sexual intercourse has declined, with 39% of 15-year-olds reporting sexual activity in 1991 compared to 15.9% in 2021; similarly, early sexual initiation decreased from 13.3% in 1991 to 4.0% in 2021 (Centers for Disease Control & Prevention, 2024; Ethier et al., 2018; Lindberg et al., 2021).

Despite these trends, contemporary frameworks and epidemiologic data suggest the need to broaden the conceptualization of early sexual initiation, encompassing a broader range of sexual behaviors, such as sexting (particularly pressured, unwanted, and coerced sexting (Van Ouytsel et al., 2022)). Sexting, defined as receiving or forwarding sexually explicit or suggestive messages and images via electronic devices or online platforms (Ngo et al., 2017), has been associated with engagement in risky sexual behaviors, such as condomless sex, a higher number of sexual partners (Dake et al., 2012), unintended pregnancy, and an increased risk of sexually transmitted infections (Rice et al., 2014). The prevalence of sexting among adolescents has increased, with 4% reporting sending and 15% receiving sexts in 2009, compared to 14.8% and 27.4%, respectively, in 2018 (Lenhart, 2009; Madigan et al., 2018; Van Ouytsel et al., 2020). These patterns in adolescent risky sexual behaviors underscore the need to expand the scope of study of early sexual initiation.

Adolescent risk-taking and early sexual initiation are influenced by multiple psychological and neurobiological factors. Particularly, the presence of externalizing disorders (i.e., attention-deficit/hyperactivity disorder [ADHD], oppositional defiant disorder [ODD], and conduct disorder [CD]) has been strongly associated with increased risk-taking, including risky sexual behaviors and early sexual initiation (Therriault et al., 2024). Moreover, externalizing disorders in adolescence are characterized by dysregulated decision-making and reward mechanisms, higher impulsivity, and difficulty with self-regulation. These characteristics, associated with heightened sensation seeking and reduced impulse control, could potentially contribute to a greater risk of early sexual initiation, multiple sexual partners, increased risk of STIs, and unintended pregnancy in this vulnerable group (Caminis et al., 2007).

As a core characteristic of externalizing disorders, impulsivity has been linked to heightened engagement in risk-taking behaviors (Martel et al., 2017; Smith et al., 1985). However, research exploring the association between impulsivity and risky sexual behaviors in this population remains limited, highlighting a critical gap in the literature. Impulsivity is a multidimensional construct characterized by rash, unpremeditated actions and difficulty delaying gratification (Curry et al., 2018). It has been conceptualized using a five-factor model (Whiteside & Lynam, 2001), which includes negative urgency (acting impulsively under negative emotions), positive urgency (acting impulsively under positive emotions), lack of planning (acting without forethought), lack of perseverance (difficulty completing tasks), and sensation seeking (desire for novel and stimulating experiences with a willingness to take risks; Lynam et al., 2006).

Studies in older adolescents and young adults, utilizing the five-factor model, suggest that impulsivity dimensions are differentially linked to specific sexual behaviors. For instance, sensation seeking is associated with engagement in casual sexual relationships, an increased number of sexual partners, and unprotected sex while under the influence of substances (Curry et al., 2018; Deckman & DeWall, 2011). Similarly, positive urgency has been linked to risky sexual behaviors, including condomless sex, multiple concurrent partners, and engagement in sex in public or unconventional settings (Zapolski et al., 2009). Additionally, previous literature has shown that sensation seeking and negative urgency are associated with early dating behaviors, which may serve as a precursor to early sexual initiation (Kwon et al., 2023). Despite these findings, research examining the association between impulsivity and early sexual initiation—including sexting and other sexual behaviors—remains scarce, particularly among young adolescents diagnosed with externalizing disorders. A previous study assessing the role of impulsivity and online sexual risk behaviors found that higher novelty seeking and risky decision-making, as well as lack of self-control, were associated with online sexual risk behaviors; nonetheless, ADHD was not a significantly associated (Chou et al., 2024). Additionally, adolescents with CD were more likely to engage in sexting than adolescents without CD (Mariamo et al., 2024).

Adolescents with externalizing disorders exhibit elevated rates of risk-taking behaviors, including early sexual initiation (Flory et al., 2006; Halkett & Hinshaw, 2021; Isaksson et al., 2018), compared to non-externalizing peers. Therefore, this study focused on a high-risk population with anticipated elevated rates of sexual behaviors and sexting compared to the general adolescent population. The present work seeks to further characterize the role of impulsivity in early sexual behaviors among these high-risk groups, with an emphasis on understanding how specific impulsivity domains contribute to engagement in sexting and early sexual initiation. Building upon prior research, we hypothesize that higher scores on impulsivity dimensions, particularly sensation seeking and negative urgency, will be associated with an increased likelihood of early sexual behaviors, including sexting, sexual intercourse, and other sexual behaviors. This study extends earlier findings that linked sensation seeking and negative urgency to early dating behaviors (Kwon et al., 2023) and aims to elucidate the broader role of the association between impulsivity and early sexual behaviors among high-risk youth. By focusing on adolescents with externalizing disorders, this research provides valuable insights into the mechanisms underlying early sexual initiation and risk-taking, contributing to the development of targeted interventions for vulnerable populations.

## Method

### Participants

This observational study included 96 participants with externalizing disorders. All met *Diagnostic and Statistical Manual of Mental Disorders* (DSM-5; American Psychiatric Association, 2013) criteria for ADHD plus either ODD, CD, or disruptive behavior disorder, unspecified. This study is a secondary data analysis that included participants that were recruited from urban and suburban areas of Indianapolis and were enrolled at ages 11–12 in an ongoing longitudinal study (data collection started in 2017) designed to characterize brain mechanisms associated with risky decision-making (Hulvershorn et al., 2013). The sample included in this study was youth aged ≥ 14 at the times of assessment of sexual behavior, although the impulsivity scale was administered at age 11–12 (baseline). As mentioned before, the current study only included participants with externalizing disorders (from an overall sample of over 200 participants which included healthy controls). We did not include participants who withdrew after the baseline measurements. Exclusion criteria for the main study included lack of English language proficiency in children, left-handedness (due to potential differences in brain lateralization and functional connectivity patterns; Willems et al., 2014), in-utero exposure to drugs, current mood disorder (based on the DSM-5 criteria, to avoid current internalizing disorders), lifetime history of psychotic symptoms, bipolar disorder, autism spectrum disorder, previous substance use, estimated full-scale IQ < 75, neurological problems, debilitating medical conditions, or contraindications to magnetic resonance imaging (MRI) scanning (these criteria were established as prerequisites for participation in the primary study involving brain imaging) (Dir et al., 2019; Kwon et al., 2021). Parental consent and youth assent were obtained prior to any assessment. Additional methods for the study and study sample have been previously published (e.g., Dir et al., 2019; Kwon et al., 2021).

### Measures and Procedure

Impulsivity at baseline was measured using the Urgency-Premeditation-Perseverance-Sensation Seeking-Positive Urgency (UPPS-P) scale for youth aged 7–13 years old (Lynam et al., 2007). Youth participants responded to 40 statements about their behavior indicating how much they agreed to the statement on a four-point Likert scale (from “not at all like me” to “very much like me”). The statements were distributed across the five different impulsivity domains of the UPPS-P which has been shown to have strong internal consistency (*α* = Cronbach’s alpha): lack of premeditation (*α* = 0.78), lack of perseverance (*α* = 0.62), negative (*α* = 0.87) and positive urgency (*α* = 0.89), and sensation seeking (*α* = 0.80; Samiefard et al., 2023). Negatively weighted items were reversed coded, and the average of the scores from each subscale comprises the UPPS-P total score (*α* = 0.89). Higher scores in each subscale denote greater symptom/trait severity.

Sexual behavioral data were collected through a self-reported, online sexual repertoire scale that was administered every 6 months once participants turned 14 years old. Observed sexual behaviors were stratified into three categories: sexting engagement, sexual intercourse, and other sexual behaviors.

Sexting engagement was assessed by four self-reported items assessing the occurrence of sexting behaviors: “Someone has sent me sexually suggestive texts or emails”, “Someone has sent me nude/semi-nude photos of themselves”, “I have sent sexually suggestive message or emails”, and “I have sent nude/semi-nude photos of myself.” Participants who responded “yes” to any of the questions were coded as having engaged in sexting behaviors at least once in their lifetime. Participants who responded “no” to all the questions were coded as having never engaged in sexting behaviors before. Engagement in other sexual behaviors (non-intercourse) was defined as engagement during the follow-up period of any of the following sexual behaviors (adapted from (Hennessy et al., 2008)), including deep kissing, touching partner’s genitals, receiving genital touch, and oral sex. Finally, sexual intercourse engagement was defined as a “yes” response to any of the following questions at any point during follow-up: “ Have you had sexual intercourse?”, “Have you had anal sex?”, and “Have you had vaginal sex?”

Confounders in the analysis were derived from baseline data and included demographic and biological characteristics, such as biological sex (male, female), family history of substance use (yes/no), and parental education as a proxy for socioeconomic status (highest educational degree attained by either parent: some or completed high school/GED, some or completed college or equivalent, and some or completed graduate degree or equivalent). Age was defined as the age at first sexual event or, for participants who did not engage in sexual behaviors, the age at the last survey assessment.

### Data Analysis

Descriptive statistical analysis was conducted to evaluate the distribution and differences of the demographic characteristics. Continuous variables were reported in means and SDs, and categorical variables were reported in frequencies (proportions), when considered suitable, according to the data distribution. Differences were assessed with the t test and chi-square/Fisher’s tests, respectively.

Unadjusted and adjusted modified Poisson regressions with robust variance were estimated to determine the relative risk between impulsivity and the three risky sexual behavior outcomes. This method allows the direct estimation of risk ratios, which prevents overestimation in comparison to odds ratios. Additionally, the model is more robust and prevents convergence problems, which makes it a more reliable method (Zou & Donner, 2013). These models were fitted in R version 4.1.0 using the generalized linear model in the “stats” package and the “sandwich” package for robust covariance matrix estimation (R Core-Team, 2021).

Missing data in the parental education (*n* = 21) were imputed by predictive mean matching (PMM; accounting for the other covariates), using the “MICE” package (Van Buuren & Groothuis-Oudshoorn, 2011). Model adjustment included predetermined confounders (sex, family history of substance use, parental education). Significance level was set at a *α* = 0.05. Multiple comparison correction was performed through a False Discovery Rate (FDR)/False Coverage-statement Rate (FCR) approach (through a permutation-based FDR point and confidence interval estimation) across all tests in the study.

## Results

A total of 96 participants with externalizing disorders, aged 14 years or older, were included in the analysis. Our study sample was predominantly male (66.7%), and most reported having at least one parent with an education equal to or higher than some college (88.0%). Approximately 42% of the youth had family members with substance use disorders. The proportion of early sexual behaviors in the sample were distributed as follows: 68% had sexted, 49% endorsed engaging in other sexual behaviors, and 25% endorsed engaging in sexual intercourse. Around 27% of the study sample reported not participating in any of the sexual behaviors measured in this study. Regarding group differences, significant differences in age (younger individuals) were found between participants who reported having engaged in sexting and other sexual behaviors compared to those who have not engaged in either behavior. No significant differences in the descriptive characteristics were found between participants who endorsed (vs not) sexual intercourse (Table 1).

**Table 1:** Descriptive characteristics^a^

	Overall Sample (*n* = 96)	Sexting (sending or receiving) Engagement			Other sexual behavior			Sexual intercourse		
		No (*n* = 31)	Yes (*n* = 65)	*p*	No (*n* = 73)	Yes (*n* = 23)	*p*	No (*n* = 72)	Yes (*n* = 24)	*p*
Age^b^	15.30 (0.92)	15.6 (0.84)	14.17 (1.44)	** < 0.01**	15.25 (0.94)	15.82 (0.96)	**0.01**	15.40 (0.96)	15.71 (0.88)	0.17
Family History SUD (yes) (%)	51 (42.5)	15 (48.4)	36 (55.4)	0.67	37 (50.7)	14 (60.9)	0.54	37 (51.4)	14 (58.3)	0.72
Sex (%)				0.13			0.15			0.55
Males	62 (66.7)	23 (79.3)	39 (60.0)		50 (71.4)	12 (52.2)		49 (69.0)	13 (59.1)	
Females	34 (33.3)	8 (20.7)	26 (40.0)		23 (28.6)	11 (47.8)		23 (31.0)	11 (40.9)	
Parents education (%)				0.33			0.59			0.30
Some or high school/GED	9 (12)	6 (19.4)	7 (10.8)		9 (12.3)	4 (17.4)		12 (16.7)	1 (4.2)	
Some or college	42 (56)	15 (48.4)	41 (63.1)		45 (61.6)	11 (47.8)		40 (55.6)	16 (66.7)	
Some or graduate degree	24 (32)	10 (32.3)	17 (26.2)		19 (26.0)	8 (34.8)		20 (27.8)	57 (29.2)	
Race				0.16			0.46			0.36
African American	30 (31.2)	11 (35.5)	19 (29.2)		25 (34.2)	5 (21.7)		22 (30.6)	8 (33.3)	
White	54 (56.2)	19 (61.3)	35 (53.8)		40 (54.8)	14 (60.9)		39 (54.2)	15 (62.5)	
Multiracial	12 (12.5)	1 (3.2)	11 (16.9)		8 (11.0)	4 (17.4)		11 (15.3)	1 (4.2)	

^a^Values mean (SD) or *n* (%) unless indicated otherwise. SUD = substance use disorder.

^b^Age at baseline (11.96 (0.52)). Age at first report on the sexual behavior or last follow-up (for naïve participants) is reported in each outcome

Significant value at* p*  <  0.05 are highlighted in bold

The study aimed to examine the association between each of the five factors of impulsivity, total UPPS scores, and sexual behaviors. Unadjusted statistically significant associations were found between sexting and overall impulsivity (UPPS-P total score; RR = 1.09, 95% CI = 1.02–1.17), lack of planning (RR = 1.26, 95% CI = 1.01–1.57), and sensation seeking (RR = 1.25, 95% CI = 1.01–1.54). Associations remained significant for overall impulsivity (ARR = 1.11, 95% CI = 1.02–1.21) and sensation seeking (ARR = 1.45, 95% CI = 1.18 -1.79), but not lack of planning, after model adjustment and multiple comparison correction (Table 2). Moreover, sensation seeking was associated with engagement in other sexual behaviors in the adjusted model (ARR = 1.44, 95% CI = 1.01–2.08). No significant associations were found between the impulsivity subscales and sexual intercourse (Table 2).

**Table 2:** Relative risks between impulsivity scores and risky sexual behaviors

	Sexting (sending or receiving) engagement		Other sexual behavior		Sexual intercourse	
	Unadjusted RR (95%CI)	Adjusted RR^a^ (95%CI)	Unadjusted RR (95%CI)	Adjusted RR^a^ (95%CI)	Unadjusted RR (95%CI)	Adjusted RR^a^ (95%CI)
Total score UPPS	**1.09** **(1.02–1.17)**	**1.11** **(1.02–1.21)**	1.03 (0.92–1.15)	1.08 (0.97–1.22)	0.97 (0.80–1.17)	0.98 (0.78–1.23)
Lack of planning	**1.26** **(1.01–1.57)**	1.28 (0.99–1.66)	0.84 (0.58–1.22)	0.97 (0.67–1.42)	0.73 (0.36–1.46)	0.79 (0.42–1.50)
Lack of perseverance	1.22 (0.85–1.77)	1.24 (0.86–1.80)	0.95 (0.56–1.62)	0.87 (0.48–1.58)	1.35 (0.58–3.15)	1.10 (0.41–2.96)
Negative urgency	1.15 (0.92–1.44)	1.09 (0.84–1.41)	1.22 (0.88–1.68)	1.35 (0.96–1.88)	1.14 (0.68–1.92)	1.18 (0.65–2.13)
Positive urgency	1.15 (0.95–1.40)	1.06 (0.87–1.30)	1.00 (0.75–1.33)	1.11 (0.85–1.45)	0.85 (0.54–1.34)	0.92 (0.56–1.51)
Sensation seeking	**1.25** **(1.01–1.54)**	**1.45** **(1.18–1.79)**	1.21 (0.88–1.67)	**1.44** **(1.01–2.08)**	0.92 (0.50–1.68)	0.92 (0.48–1.79)

^a^Adjusted models included age, sex, and parents’ education, and family history of SUDs covariates

Bold values highlight estimates with* p* < 0.05

Significant estimates after multiple correction with FDR/FCR

## Discussion

Our study examined the association between impulsivity factors and emerging sexual behaviors in a sample of young adolescents with externalizing disorders. Self-reported overall impulsivity and sensation seeking traits were associated with an increased risk of sexual behaviors, particularly sexting. Previous studies have shown that lack of premeditation, sensation seeking, and negative and positive urgency are predictors for specific risky sexual behaviors (e.g., number of partners, unprotected sex, use of substances during sex) during late adolescence and young adulthood (Curry et al., 2018). Furthermore, in young adults with externalizing disorders, negative urgency and sensation seeking are the main predictors for risky sexual behaviors (Curry et al., 2018). Consistent with this, we found that impulsivity, particularly sensation seeking, is associated with the initiation of early sexual behaviors (specifically sexting and other sexual behaviors) among adolescents with externalizing disorders. Adolescence is a particularly vulnerable stage of life, and previous research has shown that young adolescents are more likely to engage in risky sexual behaviors after participating in sexting (Houck et al., 2014). Similarly, adolescents who have engaged in behaviors such as genital touch or oral sex have a higher probability of sexual intercourse and a higher risk of risky sexual behaviors (Parti et al., 2023). While these are normative and common behaviors during adolescence, they are arguably precursors of risky sexual behaviors when engaged in during childhood and early adolescence, which can lead to significant psychosocial and health consequences (Parti et al., 2023). Therefore, these behaviors may be appropriately considered as proxies for sexual risk-taking during childhood and early adolescence (Hicks et al., 2021).

Moreover, youth and adults with more severe impulsivity are more likely to engage in sexting and other risky sexual behaviors. For example, studies with young adults have found that the exploration of posting and receiving sexual content has been significantly associated with sensation seeking (Van Ouytsel et al., 2014). While there is scarce evidence for this association in adolescents, particularly with externalizing disorders, multiple studies in adolescent clinical samples have determined a strong association between sensation seeking and risky behaviors (Dir & Cyders, 2015; Miller, 2022; Van Ouytsel et al., 2014), which align with our findings.

We speculate that the roles of sensation seeking and overall impulsivity with early sexual behaviors, particularly in high-risk youth, could possibly be explained by several neurodevelopmental factors. One reasoning follows the dual systems model which speculates a developmental imbalance during adolescence, where the socioemotional system (which drives reward seeking) matures faster than the cognitive control system (which is responsible for risk evaluation and decision-making), leading to increased risk-taking (Shulman et al., 2016). Additionally, there is the possibility that several neurobiological pathways could potentially be more sensitive during adolescence. For example, the reward circuitry is more responsive during adolescence due to the heightened activity of the mesolimbic dopamine pathway, contributing to a higher sensitivity of reward/thrill seeking (Galván, 2013), while the prefrontal cortex (responsible for executive control) is still developing, contributing to impulsive decisions and risky behaviors (Telzer, 2016).

Though we hypothesized that negative urgency was likely to be associated with sexual behaviors, which has been reported previously in the literature (Kwon et al., 2023), we found no significant associations. Negative urgency refers to the inability to regulate negative emotions, and we speculated that lower emotional regulation capacity, which could impair social connections and decision-making, would be a predictor for early sexual initiation, but our study did not reflect this association. Nonetheless, we speculate that youth who engage in other sexual behaviors are more likely to be involved in intimate relationships (Telzer, 2016), but the emergence of these behaviors is more probable during late adolescence, in comparison to our sample, which could potentially explain the results.

Although a significant association was found between sensation seeking and engagement in other sexual behaviors in the adjusted model, no associations were found for sexual intercourse, which may reflect insufficient power due to the sample size. It is also possible that the underrepresented number of events could be due to an effect of developmental timing. This sample was relatively young, and sexual intercourse often occurs later in adolescence, whereas the effect of impulsivity could potentially have been more evident in earlier more prevalent behaviors, such as sexting, compared to studies of older adolescents (Finer & Philbin, 2013). This may be best explained by the overall low engagement in sexual intercourse in our study sample, which was aged 14 years on average (i.e., 25% of the sample endorsed sexual intercourse, compared to approximately 48% of 17–18-year-old US adolescents, who endorsed sexual intercourse; Centers for Disease Control & Prevention, 2024). This is consistent with the expected initial sexual debut, which often occurs after age 15 (Magnusson et al., 2019).

Finally, preliminary unadjusted results showed additional significant associations between lack of planning and sexting. These unadjusted associations may have been confounded by covariates such as biological sex, age, family history of SUD, and parental education, as the association lost significance after adjustment. These findings aligned with the results from previous meta-analysis, which found that factors such as gender, age, and race have a moderating effect in the association between impulsivity and adolescent risky behavior (Dir et al., 2014).

The study’s results should be considered in light of its limitations. First, our study was designed as a cohort, and this secondary data analysis limited the assessment of causality and restricted the dataset to the design of the main study. Moreover, we acknowledge that our sample was limited to substance-naïve participants at baseline, thereby excluding individuals who may have already initiated substance use and could be at higher risk of early engagement in these behaviors. Second, the UPPS-P scale was assessed only at baseline, so changes in impulsivity over time were not captured, limiting our ability to infer causality. We also acknowledge that impulsivity may fluctuate during adolescence, though increases in autonomy and opportunities for risky decision-making with age could interact with these developmental changes. Third, the measurement of risky sexual behaviors was assessed on self-reported items; we acknowledge that these types of tools are prone to various types of bias. Nonetheless, it also allows for anonymity and confidentiality, and given that the behavior was assessed at multiple time points, we were able to compare the behavior through multiple assessments. Additionally, the sexting variable was simplified to overall sexting and not passive and active sexting separately due to the data imbalance and small sample (e.g., few participants reported uniquely engaging in active sexting). We acknowledge that this operationalization could potentially oversimplify the analysis, obscuring possible differences in these behaviors. Fourth, we acknowledge the results are not generalizable, as we targeted a particularly high-risk group of youth who likely warrant targeted preventive interventions. Finally, our study was limited by the sample size, affecting the power of the overall association between the impulsivity subscales and the outcomes, particularly with the engagement in other sexual behaviors and sexual intercourse. Most predictors were substantially underpowered, with achieved power well below the conventional 80% threshold, limiting confidence in the null findings for both engagement in other sexual behaviors and sexual intercourse (e.g., negative urgency 0.41 and 0.20, respectively).

Despite these limitations, this study has several strengths. This design respects temporality, as this is an ongoing longitudinal sample that is able to capture sexual initiation. This study relies on the UPPS-P impulsivity measure, a robust and valid tool in the studied age group (Lynam et al., 2007). Additionally, including sexting as a marker permitted a robust definition of early sexual behaviors, which accounts for the changing trends in sexual initiation among adolescents. This study is among the first to explore the association between impulsivity and early sexual behaviors in an adolescent sample specifically, as previous studies have overwhelmingly focused on young adults. Furthermore, our study focused on youth with externalizing disorders, an understudied population at critical risk of unsafe sexual behaviors and their deleterious consequences.

This study has important implications regarding the prevention of risky sex behaviors among the highest risk youth. Our results align with findings in a previous study, where sensation seeking was also associated with early dating (as a precursor of early sexual debut) (Kwon et al., 2023). Our findings underscore sensation seeking as an important driver for initiating sexting and other sexual behaviors, which have been associated with later risky sex behaviors. Therefore, promoting early identification and screening, as well as extending personality-targeted therapies that target youth with high sensation needs (Guillou-Landreat et al., 2021), in the sexual behavior intervention space seems warranted. In addition, the implementation of policies targeting comprehensive sexual education and the promotion of integrated mental health services within schools can ensure timely and appropriate interventions for this at-risk population. Lastly, future longitudinal studies of sexual behaviors, assessing changes in risk over time, as well as the trajectories delineated throughout adolescence, particularly in individuals with externalizing behaviors, are needed.

## Data Availability

The data used in this study are drawn from an ongoing longitudinal study following youth in the USA and will be deposited in a national data repository in 2025.

## Code Availability

The code prepared for this manuscript is available upon request.
